# Illness Perception and Medication Adherence among Adult Patients with Type 2 Diabetes Mellitus: A Scoping Review

**DOI:** 10.3390/clinpract13010007

**Published:** 2023-01-03

**Authors:** Samaher Alharbi, Aisha Alhofaian, Marym M. Alaamri

**Affiliations:** Department of Medical and Surgical Nursing, Faculty of Nursing, King Abdul Aziz University, P.O. Box 80209, Jeddah 21589, Saudi Arabia

**Keywords:** illness perception, medication adherence, treatment, glycemic control, type 2 diabetes mellitus

## Abstract

(1) Background: Type 2 diabetes mellitus (T2DM) is a global disease with a compelling impact on developed and developing economies across the globe. The World Health Organization (WHO) (2020) reported a global prevalence of 8.5% in 2014 among adults aged at least 18 years. Consequently, the condition led to a 5% increase in premature mortality from 2000 to 2016. Aim: The scoping review sought to examine illness perception and medication adherence among adult patients with T2DM. (2) Methods: The study was conducted in 2021 and covered articles published in English in the last five years. PubMed, MEDLINE, CINAHL, and ScienceDirect were the primary search engines used to generate the required scholarly records. A total of 20 studies met the inclusion criteria. (3) Results: The 20 studies selected for the scoping review covered different themes on the overall concept of illness perception and medication adherence in adults with Type 2 Diabetes Mellitus. Each study presented unique implications for research and influence on the policymaking relating to the treatment or the management of type 2 diabetes mellitus in adults of different aged groups. (4) Conclusions: The studies reveal both high and low adherence to medications in adults with type 2 diabetes mellitus. The management and treatment of the condition depend on the uptake of oral hypoglycemic agents or insulin as well as the recommended therapies to enhance the clinical outcomes of the patients.

## 1. Introduction

Type 2 diabetes mellitus (T2DM) is a global disease that has a significant impact on both developed and developing economies worldwide. The World Health Organization (WHO) reported a global prevalence of 8.5% in 2014 among adults aged at least 18 years. The prevalence of Type 2 diabetes rose from 4.5% in 1980 [1]. Consequently, the condition led to a 3% increase in mortality from 2000 to 2019. Furthermore, the WHO associates T2DM with kidney failure, heart attacks, stroke, and blindness, besides the 1.6 million deaths in 2016 due to high blood glucose [1]. The prevalence is higher in older adults and patients with high blood glucose, and other risk factors for T2DM include a lack of physical activities, unhealthy diets, high body mass index (BMI) or weight, and smoking [2].

The prevalence of T2DM has been increasing in low- and middle-income countries more than in high-income countries, which is among the risk of noncommunicable diseases (NCD) with high premature deaths in 2000–2016 [1]. This condition affects adults aged over 45 years more than young adults, teens, and children. Insulin resistance and elevated blood sugar lead to other health problems, including vision loss, heart disease, and kidney diseases. According to Khan et al. (2019) [3], T2DM affects 8529 Europeans per 100,000 of the population, and the diabetes burden is rising at a higher rate in Europe than the global average. The data show a continued rise in T2DM cases in developed nations. For comparison, Al Slamah et al. (2020) [4] noted a 7% (N  =  808) prevalence of 10,821 adults who had T2DM, which indicates an increase in diagnosed cases in Saudi Arabia and other Gulf countries. Alotaibi et al. (2017) [5] stressed that T2DM is a leading public health problem across all demographics. 

Li et al. (2020) [6] considered illness perception as the source of depressive symptoms among patients with both types of diabetes. Patients develop negative convictions about their ability to cope with the symptoms and severity of the complications arising from the condition. These perceptions determine treatment and medication adherence, which then influence clinical outcomes. Aloudah et al. (2018) [7] noted the prevalent problem of medication adherence, particularly for oral hypoglycemic agents (OHA), to manage T2DM effectively. The nature of perceptions might affect the overall adherence behaviors among patients with T2DM.

### Importance of the Review 

This study establishes an improved conceptual understanding of illness perception and medication adherence in patients with T2DM. Medication adherence and beliefs about the disease influence patients’ commitment to taking OHAs or scheduling screening. Thus, research is necessary to confirm the negative health consequences that emanate from perceptions formed during the treatment and management of T2DM. Findings from the studies might confirm the specific attitudes and behaviors towards medication protocols to enhance glycemic control across genders, age groups, and social groups. Researchers will explore the different factors contributing to positive or negative perceptions and adherence or nonadherence to T2DM medications. 

The results may help governments to understand the factors contributing to negative perceptions or nonadherence to medication and establish ways of managing the mortality and morbidity arising from T2DM. The findings could be used to inform the development of guidelines that optimize patient outcomes through multifaceted interventions rather than patient-related factors. The actions and attitudes of patients undergoing treatment or management of T2DM depend on organizational, social, and medical factors. 

The outcomes of this scoping review serve as a reference for the global healthcare system to address existing gaps in methods of reducing the global disease burden of T2DM. The prevailing perceptions and medication adherence imply the extent of severity and subsequent clinical interventions within the global health systems. Governmental and non-governmental stakeholders in the global healthcare system should create frameworks for improving illness perceptions and maintaining adherence to medication in order to reduce the burden of T2DM. Furthermore, guidelines should align with the diverse cultural, social, and economic contexts that influence the control of T2DM.

This scoping review sought to examine illness perception and medication adherence among adult patients with T2DM.

## 2. Methods

The five-step framework recommended by Arksey and O’Malley (2005) [8] was used to carry out a scoping review. The process involved: identifying the research question; identifying the relevant studies; drawing up eligibility criteria for choosing participants; mapping the extracted data; organizing, summarizing, and reporting the results.

### 2.1. Research Question 

The research question guiding this study was the following:

How do adult patients diagnose with T2DM perceive their illness and adhere to their medication? 

The Population, Intervention, Comparison, Outcome, and Time (PICOT) mnemonic was used to frame and break down the research question into searchable terms (Table 1).

### 2.2. Search Strategy 

The PICOT mnemonic generated the terms that shaped the search process of the relevant studies to answer the research question. The search terms and keywords included “illness perception”, “medication adherence”, “treatment”, “management”, “glycaemic control”, “adults”, and “type 2 diabetes mellitus.” The search terms were related to the fundamental concepts in the study, and each term highlighted the keywords the reviewer used to compile the published scholarly articles. The keywords generated specific and appropriate studies that were used in the scoping review. 

The search process required reliable electronic databases to shape the web search for the required studies. Specifically, nursing and medical databases were consulted in order to generate relevant records that addressed the research question. Online databases (PubMed, ScienceDirect, MEDLINE, and CINAHL) were accessed through the Saudi Digital Library (SDL). As each database then dictated a distinct search process, varying the search terms was imperative. The Boolean operators “AND” and “OR” were used to combine the relevant search terms for each electronic database, linking the different keywords identified through PICOT to inform the search process. The four databases expanded the scope and content of the scoping review by providing a wide range of scholarly records for selection. Table 2 outlines the keywords and search outcomes from each database.

PubMed, MEDLINE, CINAHL, and ScienceDirect were the primary search engines used to generate the required scholarly records. Inclusion and exclusion criteria were established to define the search limits and identify appropriate studies.

#### 2.2.1. Inclusion Criteria

- Records that focused on illness perceptions and medication adherence in adults with T2DM;
- Studies with adult patients aged at least 18 years;
- Full-text papers;
- Journal articles published within the last 5 years, between 2016 and 2021;
- Studies published in the English language;
- Primary sources with qualitative or quantitative research designs.

#### 2.2.2. Exclusion Criteria

- Studies with pregnant participants;
- Studies with participants under treatment for psychiatric disease;
- Studies written in languages other than English;
- Secondary sources, opinion editions, government periodicals, case reports, or lab reports.

### 2.3. Study Selection Process

The electronic databases generated 205 records after implementing the search strategy. PubMed, ScienceDirect, MEDLINE, and CINAHL produced 104, 40, 25, and 36 journal articles, respectively. Several search limits were applied, including records published in English, peer-reviewed articles, and articles published between 2016 and 2021. The researcher preferred this time limit to identify the most recent studies. The process ensured that each electronic database generated scholarly records that addressed the main research concepts.

**Figure 1:**
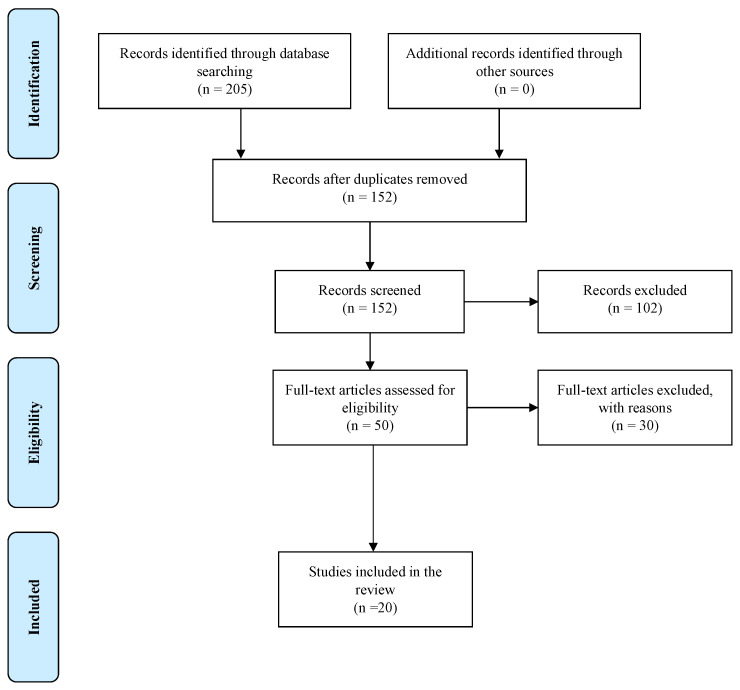
PRISMA Flow Diagram of Included Studies, adapted from Ref. [9].

After removing 53 duplicates, the reviewer screened the titles and abstracts of the remaining 152 records. The screening process then led to the exclusion of 97 articles and further removal of 5 articles based on the abstracts. The remaining 50 articles were evaluated against the eligibility criteria, and 30 articles were removed for various reasons. The remaining 20 articles were then selected for the final review. These studies addressed different standpoints on illness perceptions and medication adherence in adults with T2DM. Figure 1 outlines the PRISMA Flow Diagram that summarizes the steps followed in screening and selecting the studies for the scoping review. 

### 2.4. Data Extraction 

The selection of the studies that aligned with the inclusion criteria was followed by a manual screening of the studies and subsequent identification of the specific characteristics of each research article. The data extraction table in Supplementary SA outlines the specific details of each scholarly record, such as author(s), publication date, country of study, research design, sample, participants, intervention groups, control or placebo, and comparison groups. The review matrix included follow-up, the different outcome measures, findings, and additional comments about the reliability of the research. The data extraction process facilitated the assessment of the overall quality of the outcomes and validity of each research study. A rigorous selection of the primary sources was imperative to ensure that the scoping review utilized studies with high validity.

## 3. Findings/Results

### 3.1. Description of the Studies 

The 20 studies selected for the scoping review covered different themes on the overall concept of illness perception and medication adherence in adults with T2DM. The studies deliberated on the prevalent problem facing patients with T2DM and justified the extent of the problem with evidence on, for instance, negative perceptions of OHAs and subsequent inconsistency in using the medication to enhance glycemic control. The studies further relied on different study designs to gather and analyze evidence from various settings as well as participant groups. Each study presented unique implications for research, nursing practice, and influence on the policymaking relating to the treatment or the management of T2DM in adults of different aged groups. 

The studies deployed different methods and designs to collect insights on illness perception and medication adherence in adult patients with T2DM. The scoping includes 20 quantitative studies [10,11,12,13,14,15,16,17,18,19,20,21,22,23,24,25,26,27,28,29]. Among the quantitative research, we found one randomized controlled trial (RCT) and two retrospective studies, while the remaining were cross-sectional studies. 

The studies provided different levels of evidence, as outlined in Table 3. This shows that the authors conducted studies that sought the highest level of evidence to enhance the overall reliability and validity of the respective outcomes. The RCT gathered the highest level of evidence, while the remaining 16 studies had relatively high-level evidence that further contributed to the reliability of the results in answering the research question framed for the scoping review. 

Regarding the countries in the selected studies, most studies were conducted in Saudi Arabia (4). The remaining studies were conducted in the USA (2), China (2), Malaysia (2), Finland (1), Taiwan (1), Pakistan (1), Japan (1), Lebanon (1), Ghana (2), Bangladesh (1), Iran (1), and Singapore (1). 

The sample size ranged from 30 to 24,192 in the quantitative studies. All studies included male and female individuals with T2DM: male range (27–12.216) and female range (39–11.976). The mean age ranged from 44.7 years (SD = 15.6) to 65.8 years (SD = 10).

### 3.2. Quality Assessment 

Two expert reviewers used the quality scoring system tool developed [30] as cited in [31]. This tool was used to assess the methodological rigor of the selected studies. It contains nine items that evaluate the abstract, reported method, sampling, analysis, ethics, bias, generalizability, and implications using the scoring system of “good”, “fair”, “poor”, or “very poor”. The results of the assessment are shown in Supplementary SB.

## 4. Discussion

### 4.1. Themes from the Chosen Studies

Different compelling themes emerged from the study to underscore the overall concept of illness perception and medication adherence in adult patients with T2DM. The authors discussed illness perceptions in line with the management, self-care practices, and treatment of T2DM. The studies further discussed medication adherence in isolation and linked it to patients’ health beliefs, social support, and personal convictions, as well as the severity of the disease. Moreover, the studies present illness perception as the antecedent for medication adherence or nonadherence in adult patients with T2DM. The studies differed on the relationship between medication adherence and perceptions towards T2DM. 

### 4.2. Theme 1: Illness Perception towards T2DM 

Several studies showed that health literacy and medication beliefs shaped the perception of illness among adults aged at least 20 years. Shiyanbola et al. (2018) [10] affirmed the link between the formation of perceptions and health literacy. The attainment of adequate literacy levels alongside medication beliefs shaped perceptions regarding the self-efficacy associated with OHAs or insulin in 174 participants. The mean age of the participants was 58.7 (SD = 12.8) years; 57.5% were female, 67.8% were non-Hispanic white, and 24.7% were African American.

Lee et al. (2016) [11] established that empowerment perceptions form when health literacy aligns with self-efficacy, self-care behaviors, as well as knowledge or beliefs about medication, as argued by Shiyanbola et al. (2018) [10]. While the study used a bigger sample size, it demonstrated the moderating effect of health literacy and empowerment perceptions in influencing self-care behaviors in 295 patients with T2DM with a mean age of 58.2 years (SD 11.8), and 57.3% of them were male. This indicated that empowerment perceptions exist alongside awareness about the condition and self-care initiatives to reduce complications; furthermore, the provision of the right environment should aid in building patient compliance with T2DM management. The role of diabetes knowledge and illness perceptions in shaping self-care practices for managing T2DM emerged in another study by Kugbey et al. (2017) [12]. This cross-sectional survey of 160 participants confirmed that illness perception creates self-care behaviors to reduce further complications of T2DM. The two factors predicted patients’ diet, exercise, blood sugar testing, and diabetes foot care for illness control. Therefore, illness perception forms well when patients across age groups and genders develop a sufficient understanding of diabetes. 

Education and income level play an important role in determining patient perceptions about their illness. In a study by Shiyanbola et al. (2018) [10] in the USA, patients with a high school degree or higher and an income equal to or more than USD 20,000 reported a low threatening illness perception. Conversely, Iranian patients with high school education and moderate-income reported slightly higher negative perceptions of T2DM (Bilondi et al., 2021) [13].

Koponen et al. (2016) [14] observed that patients perceived autonomous motivation, support, and self-care competence drive how often they engage in physical activities. The study sought insights from 2866 participants with a mean age of 63 years, and 55.7% were male. Further, Kugbey et al. [12] determined that the maintenance of a consistent physically active lifestyle created perceptions of recovery from the disease among their sample. However, Hashimoto et al. (2019) [15] established that disease knowledge, clinical characteristics, and demographics underline self-care management initiatives to reduce T2DM complications. 

Illness perceptions influence the management and treatment of T2DM by shaping patients’ behaviors and actions. From a convenience sample of 30 Saudi patients with T2DM; (60%) were male, Albargawi et al. (2016) [16] established that doctors influenced diabetes management in 95% of the participants, with patients registering high levels of self-efficacy and uptake of diabetes management initiatives. Perceptions about godly influence as the health locus of control also led to the uptake of a specific diet and proper foot care. Female participants showed more adherence to self-care practices than their male counterparts. Perceptions emerged as a moderator of exercise, dietary intake, foot care management, and overall self-efficacy towards managing T2DM despite relying on a small sample that affected the generalizability of the findings. However, Farhat et al. (2019) [17] relied on a larger and more representative sample of 207 patients to affirm the role of perceptions in shaping QOL and subsequent adherence to T2DM medications. The mean age of patients was 53.19 ± 9.24 years, and males consisted approximately 53.7% of the sample. The perceived effort of the management process translates into increased physical exercise, psychological health, a better environment, and relationships. The outcomes observed by Farhat et al. (2019) [17] and Albargawi et al. (2016) [16] affirm the link between illness perceptions and medication adherence in adults with T2DM.

### 4.3. Theme 2: Medication Adherence towards T2DM 

Different health beliefs about T2DM determine the level of adherence to medications. Alatawi et al. (2016) [18] found that adherence depended on the beliefs towards the prescriptions and potential T2DM complications. The cross-sectional study analyzed the data based on health belief models to understand self-reported health beliefs and medication-taking behaviors in Saudi Arabia. Age and educational level determined beliefs about the effectiveness of oral options and insulin in 58% of the 220 Saudi adult patients with a mean age of 52.1 ± 11.3 years. The gaps led to 60% failure to take medicine according to the prescribed time and interval. The low adherence to OHAs and insulin affirmed the perceived medication benefits, susceptibility, and self-efficacy as causes of poor medication-taking behaviors. 

In contrast, He et al. (2017) [19] focused on adherence to insulin rather than on OHAs to understand the overall persistence of 24,192 Chinese patients with a mean age of 58.9 ± 11.5 and 49.5% being female. The findings supported the outcomes of Alatawi et al. (2016) [18] in terms of general poor adherence to medication, despite severe hypoglycemic events, hypertension, and dyslipidemia among the patients. As over 53% of the Chinese patients showed reluctance to take the medication, this study shows that patients may be unwilling to persist with the intake of human insulin as initial therapy. This is because of analog-insulin’s improved physiologic time-action profiles, a lower risk of developing hypoglycemia, and more flexible dosing.

The adherence to the medications for T2DM depends more on the self-care activities among patients than the beliefs conceptualized by [18]. Jannoo and Khan (2018) [20] established self-care activities as predictors of medication adherence after examining 497 Malaysian patients, comprising 53.7% men and 46.3% women. The outcomes from the Morisky Medication Adherence Scale and the Summary of Diabetes Self-Care Activities showed subjects with an average age of 55.5 recorded low adherence. Adherence was also shown to vary with ethnicity, as 47.7% of Malays, 34.8% of Indians, and 17.5% were Chinese; the study recorded poor self-care behaviors of seeking glycemic control and subsequent failure to take insulin or OHAs. The patients were on a poor diet and recorded low BMI and HbA1c levels. Butt et al. (2016) [21] confirmed the link between poor HbA1c and low medication adherence but with a smaller sample size of 73 patients from Malaysia; 57.6% were Malays, 22.7% were Chinese, and 19.7% were Indians. The mean age of participants was 57.2 ± 10.78 years, and the female was (59.1%). The study further defined the effectiveness of self-care behaviors based on the clinical outcomes, such as the quality of life (QOL) of Malaysian patients. Notably, participating in a pharmacist-led diabetes intervention program showed significant changes in medication adherence between the control and intervention groups.

Findings from Farhat et al. (2019) [17] revealed further predictors of medical nonadherence among Lebanese adult patients with T2DM while also exploring QOL similar to [21]. The 207 diabetic patients who were taking oral glucose-lowering drugs (OGLD) registered varied treatment and medication adherence, as they were dependent on QOL as well as the perception of the condition. BMI, treatment satisfaction, and physical health underlined the QOL, which created the perceived effectiveness of OGLDs in managing T2DM. Furthermore, offering educational intervention enhanced awareness about T2DM medications. Therefore, poor medication adherence should be low, as suggested by [18,21], due to the varied nature of the perceived effectiveness of the medication. 

Hashimoto et al. (2019) [15] agreed that patients’ perceptions are the primary determinant of medication adherence following a cross-sectional study of 157 Japanese patients; they had a mean age of 65.8 (11.8), and 69.2% of them were men. The Japanese study defined different determinants of medication adherence, such as BMI, family history of T2DM, and diabetes knowledge. Therefore, the context and circumstances of adult patients shape their uptake of medications and treatment. In the study conducted by Islam et al. (2021) [22], the factors influencing T2DM patients’ medication adherence were evaluated, and it was found that 46.3% of 2070 patients had suboptimal levels of medication adherence. The mean age of the participants was 50.2 ± 10.2 years (56.2% females). Patients with a family history of diabetes were more likely to adhere to their anti-diabetic drugs, which indicates that they have more knowledge about diabetes from their family members and receive more supportive behaviors, leading to greater motivation.

Adherence to medication is poorer in young adults of various ethnicities than in older adults. Lee et al. (2017) [23] noted rampant nonadherence among 57.1% of the 382 sampled Asian young adults in China and Singapore during a cross-sectional study, The mean age was 62 ± 10.4 years, and 53.4% were female. Specifically, patients registered low medical adherence to OHAs and subsequent glycemic control despite undergoing polytherapy treatment. Therefore, in the case of young adults, daily medications appear to exacerbate medication non-adherence. While the elderly appear to have higher levels of compliance due to social and psychological support, family support is crucial and could enhance medication adherence by reminding the patient of their medication regimen.

AlQarni et al. (2019) [24] carried out a cross-sectional study of 212 Saudi adults with a mean age of 44.17 ± 15.6 years, and most of the patients were male (67%). The finding was that two-thirds of patients did not consistently take their prescribed anti-diabetic medications. The study also showed that older patients were more likely to be adherent, and this may be because older patients experience comorbidities and therefore tend to have a larger number of medicines included on their prescription. However, Nazir et al. (2016) [25] suggested that disease-related knowledge is the primary driver of medication adherence and subsequent glycemic control following a study of 392 Pakistanis with T2DM. The mean age of patients was 50.77 ± 9.6 years, with 56.6% being males. Shiyanbola et al. (2018) [10] agreed with Nazir et al. (2016) [25] on the effects of health literacy in moderating perceptions and adherence to medication; this was observed for more women than men and more White participants than participants of other races. Self-efficacy scores, health literacy, and overall beliefs shaped both adherence and the pursuit of treatment. In general, studies presented differing views of medication adherence and moderating factors among adult patients with T2DM.

### 4.4. Theme 3: Association between Illness Perceptions and Medication Adherence 

A variety of factors shape the perception of illness, which may then lead to either adherence or nonadherence to T2DM medications. Farhat et al. (2019) [17] noted patients’ adherence to OGLD was dependent on one’s QOL, treatment satisfaction, and overall perceived efficacy in 207 patients. Female gender and high QOL of life scores shaped positive perceptions and adherence more than male gender or low QOL. However, any perceptions about the side effects, psychological impact, and global satisfaction of medication distorted the treatment as well as subsequent management of T2DM. Kretchy et al. (2020) [26] found that 66.5% of 188 patients had poor medication adherence, with a mean age of 59.3 ± 11.9 years and a female predominance of 72.3%. The study also revealed that T2DM patients were concerned about the quality of their physicians’ care and social support. Thus, patients with poor medication adherence have uncomfortable social conditions, feelings of isolation and anger, and discouragement with T2DM treatment.

Albargawi et al. (2016) [16] suggested that creating a proper health locus of control in addition to self-efficacy could support adherence to oral drugs and insulin-based treatment, based on their sample of 30 Saudi T2DM patients. In this study, perceptions about godly influence and doctors’ recommendations improved adherence to medication as well as levels of physical exercise, foot care performance, and specific dietary intake. Although there were implications for the building of perceptions through awareness campaigns, the generalizability of the Albargawi study was low. Despite this, the study suggests that positive perceptions of T2DM-related health can create high adherence to oral medication and insulin and promote self-care management initiatives. 

In the study of 115 adults with T2DM with a mean age of 56 years (SD 12.43) and 58% of them were male, Alyami et al. (2019) [27] determined that 69% of participants had poor adherence to medication, alongside sub-optimal glycemic control. Patients who simply focussed on the consequences of the illness were less inclined to adhere to their medication schedule, while those who understood the disease were more likely to comply with the medication regimen. Optimal adherence was found in patients who recognized that diabetes could be controlled through medication and had fewer issues with taking medicine. Additionally, Bilondi et al. (2021) [13] report that patients who have a positive perception of their illness and control their health condition are likely to have high medication adherence. This study investigates the relationship between illness perception and adherence to medication among 260 Iranian patients with T2DM with a mean age of 59.05 ± 11.55 years, and 59.23% were female.

Patient characteristics can also shape the perceptions that inform their adherence to medications for treating or managing T2DM in adult patients. Hashimoto et al. (2019) [15] performed a principal component analysis and cluster analysis of 157 patients to understand their perceptions of access to proper medical treatment and the status of medicine intake, which the authors indicate built their commitment to the prescribed protocols. The patients’ characteristics, such as diabetes family history and knowledge, moderated patients’ perceptions about drug efficacy. Consequently, the impact of perceptions on the medication adherence patterns varied among patients along with BMIs, family history of the condition, and orderly living. He et al. (2017) [19] established that adherence to or persistence with insulin therapy depended more on the patients’ perceptions of the drug than their overall knowledge about the disease. Consequently, the initiation with premixed and analog insulin led to the acceptance of insulin therapy as well as a follow-up, as 51.9% of the 24,192 Chinese patients reported they understood their impact on their health. Furthermore, the existence of comorbidities such as hypertension and dyslipidemia led patients to seek medication interventions more often. 

Illness perceptions enhance the uptake of oral or insulin therapy when patients gain sufficient diabetes knowledge and adopt self-care practices for T2DM. According to Kugbey et al. (2017) [12], their sample of 160 participants with a mean age of 60.3 years (SD 12.04) and 57.3% being male from Ghana acknowledged the influence of diabetes knowledge on their perceptions about the efficacy of the T2DM treatment protocols. Positive perceptions led to the adoption of self-care practices, such as exercise, blood sugar testing, and a healthy diet. The patients also relied on the knowledge to perform foot care. However, appropriate psychosocial interventions and awareness campaigns are needed to influence patients’ ability to uphold practices that inform the uptake of insulin or OHAs besides self-care practices. This assertion was also reflected by Lee et al. (2016) [11] when they noted the value of empowerment perceptions as the trigger for self-care behaviors and glycemic control among adult patients with T2DM. Health literacy from healthcare providers was more impactful than an authoritative initiative to build patient compliance with the medication for glycemic control. The self-care behaviors led to the better management of HbA1c irrespective of patient’s age, gender, or duration of T2DM, which further underlined high adherence to medications for managing or treating T2DM or its comorbidities. 

Negative perceptions towards injection devices lead to poor medication adherence. Matza et al. (2018) [28] agreed that knowledge about the treatment process besides the medication is the primary driver of positive perceptions towards their intake. In Matza et al. (2018) [28] study, 404 patients receiving treatment with liraglutide and dulaglutide indicated overall acceptance of injection devices and subsequent use of the medications for managing their condition. The majority of study participants were white (79.2%), followed by African Americans (14.6%) and other races (7.8%), with a mean age (SD) of 60.7 (11.4) years. Overall, beliefs and knowledge shaped their perceptions towards the management or acceptability of medication for T2DM. The patients expressed an increased preference for the devices due to their beliefs about their treatment efficacy. The study concentrated on the devices rather than the actual medications, such as insulin or OHAs. 

However, Nie et al. (2018) [29] found from 304 patients that illness and risk perceptions emanated from health literacy. The illness-related perceptions and risks associated with T2DM led to health-promotion self-care behaviors among Chinese patients. The effort of creating the right knowledge and informing various health-oriented behaviors reduces diabetes complications among adult patients. 

According to Shiyanbola et al. (2018) [10], adults aged 20 years and above are more likely to form positive perceptions about T2DM due to health literacy, mainly numeracy. The findings from the analysis of 174 patients confirmed that literacy eliminated beliefs and the subsequent threatening nature of T2DM or its comorbidities. Elimination of the concerns led to better adherence to the medications, irrespective of age or gender. Lee et al. (2016) [11] supported using health literacy to create awareness of the disease and encourage self-care practices for proper glycemic control. Patients’ knowledge about the disease improves medication adherence by eliminating cultural, social, or personal convictions about T2DM or its complications. Consequently, literacy builds patient compliance with the treatment or management of the condition.

## 5. Limitation 

While reviewing the literature, we discovered several limitations in the search strategy when applying keywords—the search terms—since there were few studies, systematic reviews, and meta-analyses to be found on this topic, both locally in Saudi Arabia and globally. As far as we can determine, this review is one of the only comprehensive reviews available to researchers and interested parties. Secondly, since the scope of the search was restricted to recent studies, our findings are not generalizable, and we are unable to reach solid conclusions or offer recommendations. 

## 6. Conclusions 

The critical analysis of the 20 selected studies revealed a range of levels of adherence to medication in adults with T2DM. The management and treatment of the condition depend on the uptake of OHAs or insulin as well as the recommended therapies to enhance the clinical outcomes of the patients. On the other hand, the studies agree persistent illness perceptions exist of T2DM or devices for injecting the medications. Accordingly, some authors considered the influence of medication and the severity of T2DM or its comorbidities on illness perception. The studies also considered the personal beliefs and knowledge gathered on T2DM as a predictor of medication adherence or self-care practices. The findings show that participation in practices such as blood sugar testing, specific diets, avoiding smoking, participating in physical exercise, and foot care performance influence the adoption of the prescribed treatment or management programs. Indeed, illness perception leads to medication adherence, which might vary according to one’s knowledge of T2DM, gender, and age. Overall, the studies indicate older adults and women adhere to medications more than younger adults and male patients. 

## Figures and Tables

**Table 1:** Framing Research Question with PICOT Framework.

PICOT	SEARCH TERMS	PICOT QUESTION
Population	Adult patients	How do adult patients diagnosed with T2DM perceive their illness and adhere to their medication?
Intervention	Diagnosed with T2DM	
Comparison	Nonapplicable	
Outcome	Perceive their illness and adhere to their medication	
Time	Nonapplicable	

**Table 2:** Keywords, Boolean Operators, Electronic Databases, and Records Found.

Keywords	Boolean Operators	Electronic Databases (Via SDL)	Records Found
“Illness perception”, “medication adherence”, “treatment”, “management”, “glycaemic control”, “adults”, “type 2 diabetes mellitus”	“AND” “OR”	PubMed	104
		ScienceDirect	40
		MEDLINE	25
		CINAHL	36

**Table 3:** Levels of Evidence for the 20 Studies.

Level of Evidence	Research Design	Studies
I	RCTs; systematic reviews; meta-analysis	Butt et al. (2016) [21]
II	Quasi-experimental studies	-
III	Nonexperimental studies	Alatawi et al. (2016) [18] Matza et al. (2018) [28] Albargawi et al. (2016) [16] Koponen et al. (2016) [14] Shiyanbola et al. (2018) [10] Nie et al. (2018) [29] Lee et al. (2016) [11] Kugbey et al. (2017) [12] Hashimoto et al. (2019) [15] Farhat et al. (2019) [17] Lee et al. (2017) [23] Nazir et al. (2016) [25] Jannoo & Khan (2019) [20] He et al. (2017) [19] Alqarni et al. (2019) [24] Alyami et al. (2019) [27] Kretchy et al. (2020) [26] Islam et al. (2021) [22] Bilondi et al. (2021) [13]
IV	Expert opinions based on scientific evidence	-
V	Case reports	-

- Not applicable.

## Data Availability

Not applicable.

## Supplementary Materials

The following supporting information can be downloaded at: https://www.mdpi.com/article/10.3390/clinpract13010007/s1, Supplementary SA: Literature Review Matrix. Supplementary SB. Quality Assessment of Included Studies.
