# Supplementary material for: Illness Perception and Medication Adherence among Adult Patients with Type 2 Diabetes Mellitus: A Scoping Review
Source: Clin Pract. 2023 Jan 3;13(1):71–83. doi: 10.3390/clinpract13010007 (PMC9844476; doi:10.3390/clinpract13010007)
Supplement: Supplementary file 1 [file clinpract-13-00007-s001.zip › Supplementary A .pdf]

Supplementary A: Data Extraction Table

Study Reference	Country of Origin	Total Sample	Study Design	Types of Participants	Intervention Group	Control/ Placebo/ Comparison Group	Duration of Intervention	Follo w Up	Outcome Measures	Main Findings	Additional Comments
Bilondi et al. (2021)	Iran	260	cross-sectional study	Adults with T2DM	-	-	-	-	the rela-tionship between illness perception and medication adherence in patients with T2DM.	r illness perception as moderate and low medication adherence	The study is valid and highly reliable.
Kretchy et al. (2020)	Ghana	188	cross-sectional study	Adults with T2DM	-	-	-	-	distress associated with T2DM and to examine its association with medication adherence	44.7% of the patients recorded high levels of diabetes-related distress. 66.5% of had Poor	The study is valid and highly reliable.

										adherence to medications	
Islam et al. (2021)	Bangladesh	500	cross-sectional study	Adults with T2DM	-	-	-	-	T2DM medication adherence and factors associated with low adherence	42.8% of participants reported low medication adherence, age was negatively associated with low adherence	The study was valid and highly reliable as well as generalisable.
Alatawi et al. (2016)	Saudi Arabia	220	Cross-sectional design	Adults with T2DM aged 20–79 years	-	-	-	-	T2DM medication adherence, medication-taking behaviours, health beliefs, and complications	58.0% of the responders were on oral drugs and insulin, and<br>16.7% had knowledge of HbA1c.<br>40.0% had low adherence to the	The study was valid and highly reliable as well as generalisable.

										medication due to time and interval of the medications.	
Albargawi et al. (2016)	Saudi Arabia	30	Descriptiv e correlation design	Arab-speaking adults with T2DM	-	-	-	-	Perceptions towards T2DM<br>medication adherence and<br>self-efficacy	53.0% of self-efficacy<br>perceptions emerged from godly beliefs.<br>The perceptions shaped health locus of control and medication adherence.<br>Self-care management informed the overall medication adherence.	The study is transferable and applicable to Arab-speaking adults with T2DM and healthcare systems.
AlQarni et al. (2019)	Saudi Arabia	212	cross-sectional study	Adult patients with T2DM	-	-	-	-	T2DM medication adherence	The patients showed low medication adherence	The study was valid and highly reliable as well as

											generalisable .
Alyami et al. (2019)	Saudi Arabia	115	cross-sectional study	Saudi patients with T2DM, in mean age 56±12.43 years (58% male)	-	-	-	-	Illness Perceptions, HbA1c, And medication Adherence	69% patients reported poor medication adherence	
Butt et al. (2016)	Malaysi a	73	RCT	T2DM patients attending endocrine clinic	The intervention group (*n* = 36) received guidance and education from pharmacists	The control group (*n* = 37) did not experience intervention and education but received standard care	3–6 months	-	Morisky scores and QOL on the prevalence of T2DM after HbA1c and overall medication adherence	The intervention group registered increased Morisky scores and QOL, which then led to medical adherence. No significant change was seen in the control group.	The study shows high validity and is replicable in other studies.
Farhat et al. (2018)	Lebanon	207	Cross-sectional study	Adult patients with T2DM	-	-	-	-	Effectivenes s score, QOL score,	High effectiveness and QOL scores	The outcomes are generalisable

									BMI, global satisfaction, social relationships, and psychological health	affected adherence to medications. The study considered awareness campaigns as effective.	to the adults with T2DM.
Hashimoto et al. (2019)	Japan	157	Cross-sectional study	Patients with T2DM a history of diabetes	-	-	-	-	The link between perceptions of patients with T2DM and medication adherence	The perception of an orderly life led to poor medication adherence. Diabetes knowledge and disease determined medication adherence.	The study used a replicable methodology.
He et al. (2017)	China	24,192	Retrospective database analysis	Adults with T2DM who underwent insulin therapy	-	-	-	-	Insulin resistance and persistence to the insulin therapy	The patients registered low persistence and adherence to the insulin therapy due	The study is transferable to patients with T2DM after using a large representative sample.

										to the negative perceptions towards premixed, basal, and prandial insulin.	
Jannoo and Khan (2019)	Malaysia	497	Survey	Adult patients with T2DM aged at least 18 years	-	-	-	-	Medication adherence,<br>self-care activities,<br>risk factors, and self-care behaviour	The patients showed moderate medication adherence and low observation of blood sugar testing.	The study is valid and highly reliable.
Koponen et al. (2016)	Finland	2,866	Cross-sectional study	Adult patients with T2DM with a mean age of 63 years	-	-	-	-	Physical activity,<br>perceived autonomous support, motivation, and self-care competence	Self-care competence increased with perceived autonomy in motivation and seeking support.<br>Those who participated in physical activities	The study is replicable and generalisable to the patients with T2DM anchoring their treatment on self-care competence, such as

										reported better adherence to treatment and management of T2DM.	physical exercises.
Kugbey et al. (2017)	Ghana	160 participants	Cross-sectional study	45 males and 115 female adults with T2DM	-	-	-	-	Illness perception and diabetes knowledge,<br><br>medication adherence and self-care practices	Diabetes knowledge led to increased self-care practices, such as specific diet, exercise, and performance of self-care.<br><br>Illness perception increased and enhanced blood sugar testing and foot care after acquiring knowledge on T2DM.	The study is highly valid and reliable to different patients in middle-income countries.

Lee et al. (2016)	Taiwan	295	Cross-sectional study	Patients with T2DM attending endocrine clinics	-	-	-	-	Modifying self-care behaviour and empowerment perceptions and health literacy	Empowerment perceptions emerged from health literacy and subsequent self-efficacy. Self-care behaviour led to improved glycaemic control. Healthcare providers offer health literacy.	The study is generalisable and effective addressing perceptions towards diabetes in the healthcare system.
Lee et al. (2017)	Singapore	382	Cross-sectional survey	Adult patients with T2DM in primary care outpatient clinic	-	-	-	-	Medication adherence, associated factors with T2DM	Young adult participants registered low perceptions towards T2DM, which then led to poor glycaemic control after attending polytherapy.	The study is generalisable to Asian communities seeking to influence uptake of therapy and medication for managing T2DM.

Matza et al. (2018)	USA	404	Cross-sectional study	Adult patients with T2DM in 49 states	-	-	-	-	Perceptions towards injection devices for T2DM medicine, scores on liraglutide and dulaglutide injection devices	The study established poor acceptance to T2DM due to the negative perceptions of liraglutide and dulaglutide devices.	The studies are valid and reliable.
Nazir et al. (2016)	Pakistan	392	Cross-sectional analysis	Adult patients with T2DM	-	-	-	-	Diabetes-related knowledge, treatment adherence, glycaemic control among adult patients with T2DM	Disease-related knowledge assisted in the increase in glycated haemoglobin levels despite the duration of the disease across participants of varied age groups and genders.	The study is generalisable and transferable to older adults aged at least 50 years.

Nie et al. (2018)	China	304	Cross-sectional study	Chinese patients with T2DM	-	-	-	-	Patient characteristics,<br><br>illness perceptions, future risk perceptions,<br><br>health-promoting self-care behaviour within 6 months	Increased illness and risk perceptions on T2DM led to health-promoting self-care behaviour.<br><br>Tailoring interventions to meet the needs of diabetic patients increased adherence to treatment in Chinese patients.	The study offers a replicable methodology and transferable findings to Asian communities with T2DM cases.
Shiyanbola et al. (2018)	USA	174	Cross-sectional study	Adults with T2DM aged 20 years and over	-	-	-	-	Beliefs about medicine,<br><br>family medicine clinics and illness perceptions,	Beliefs about medicine and increased literacy and self-efficacy affected adherence to the medicine.<br><br>Adherence to the OHAs or	The study is generalisable to U.S. patients and other advanced economies with T2DM as part of

	numeracy and document literacy	insulin increased due to the self-care initiative.	public health problem.

Note. T2DM = Type 2 Diabetes Mellitus
