# Supplementary material for: Illness Perception and Medication Adherence among Adult Patients with Type 2 Diabetes Mellitus: A Scoping Review
Source: Clin Pract. 2023 Jan 3;13(1):71–83. doi: 10.3390/clinpract13010007 (PMC9844476; doi:10.3390/clinpract13010007)
Supplement: Supplementary file 1 [file clinpract-13-00007-s001.zip › Supplementary B.pdf]

Supplementary B. Quality assessment of included studies.

Author(s), year of publication	Abstract and title	Introduction and aims	Method and data	Sampling	Data analysis	Ethics and bias	Findings/ results	Transferabil ity/generaliz ability	Implications and usefulness	Total scoring /36
Alatawi et al. (2016)	4	4	3	4	3	4	4	4	4	34
Albargawi et al. (2016)	4	4	4	4	4	4	4	4	4	36
Butt et al. (2016)	4	4	4	4	4	4	4	3	4	35
Farhat et al. (2018)	4	4	4	4	4	3	4	3	4	34
Hashimoto et al. (2019)	4	4	4	4	4	4	4	3	3	34
He et al. (2017)	4	4	4	4	4	4	4	4	4	36
Jannoo and Khan (2019)	4	4	4	3	4	2	4	4	4	33
Koponen et al. (2016)	4	4	4	4	4	4	4	3	4	35
Kugbey et al. (2017)	4	4	4	3	3	4	4	4	4	34
Lee et al. (2016)	4	2	3	3	4	4	4	4	4	32
Lee et al. (2017)	4	4	4	4	3	3	4	4	3	33
Matza et al. (2018)	3	3	4	4	3	4	4	4	3	32
Nazir et al. (2016)	4	4	4	3	4	4	4	3	3	33
Nie et al. (2018)	4	4	3	3	4	4	4	4	4	34

Shiyanbola et al. (2018)	3	4	4	4	4	4	4	4	4	35
AlQarni et al. (2019)	4	4	4	3	4	4	4	4	4	35
Alyami et al. (2019)	4	4	4	3	4	2	4	4	4	33
Kretchy et al. (2020)	4	4	4	4	4	3	4	3	4	34
Bilondi et al. (2021)	4	4	4	4	4	4	4	3	4	35
Islam et al. (2021)	4	4	4	3	3	4	4	4	4	34
